# The impact of patient advisors on healthcare outcomes: a systematic review

**DOI:** 10.1186/s12913-017-2630-4

**Published:** 2017-10-23

**Authors:** Anjana E. Sharma, Margae Knox, Victor L. Mleczko, J. Nwando Olayiwola

**Affiliations:** 1 Center for Excellence in Primary Care, Department of Family & Community Medicine, UCSF, 995 Potrero Ave, Ward 83, San Francisco, CA 94110 USA; 2 Contra Costa Regional Medical Center, Family Medicine Residency Program, 2500 Alhambra Avenue, Martinez, CA 94553 USA; 3 University of California, San Francisco, 2120 University Avenue, Berkeley, CA 94704 USA

**Keywords:** Patient engagement, Patient advisory councils, Experience of care, Practice improvement

## Abstract

**Background:**

Patient advisory councils are a way for healthcare organizations to promote patient engagement. Despite mandates to implement patient advisory councils through programs like the Patient-Centered Medical Home (PCMH), there is a paucity of data measuring the impact of patients functioning in advisory roles. Our objective is to investigate whether patient engagement in patient advisory councils is linked to improvements in clinical quality, patient safety or patient satisfaction.

**Methods:**

We searched PubMed, SCOPUS, CINAHL and Google Scholar for English language publications between November 2002 to August 2015, using a combination of “patient advisor” and “care outcomes” search terms. Article selection utilized dual screening facilitated by DistillerSR software, with group discussion to resolve discordance. Observational studies, randomized controlled trials, and case studies were included that described patients serving in an advisory role where primary outcomes were mentioned. Reference lists of included studies and grey literature searches were conducted. Qualitative thematic analysis was performed to synthesize results.

**Results:**

Database searching yielded 639 articles total after removing duplicates, with 129 articles meeting full text inclusion criteria. 32 articles were identified for final review, 16 of which were case studies. Advisory roles included patient advisory councils, ad-hoc patient committees, community advisory councils, experience-based co-design, and other. Four practice-based studies from one research group, involving community advisors in the design of public health interventions, found improved clinical outcomes. No prospective experimental studies assessed the impact of patient advisors on patient safety or patient satisfaction. One cluster-randomized RCT showed that patient advisors helped health care planning efforts identify priorities more aligned with the PCMH. Ten case studies reported anecdotal benefit to individual patient advisors.

**Conclusion:**

Five included studies demonstrate promising methods for evaluating patient engagement in healthcare delivery and describe impacts on clinical outcomes and priority setting. Based on the case studies found, patient advisors tend to contribute to patient-facing services that may affect clinical care but are not easily evaluated. As clinics and hospitals implement patient advisory councils, rigorous evaluation of their programs is needed to support the expansion of system-level patient engagement.

**Trial registration:**

This systematic review was registered in the PROSPERO database of the University of York Centre for Reviews and Dissemination (ID: 2015:CRD42015030020).

**Electronic supplementary material:**

The online version of this article (10.1186/s12913-017-2630-4) contains supplementary material, which is available to authorized users.

## Background

Patient engagement can be defined as active partnership between patients, families and caregivers working together to improve healthcare delivery [1]. Patient engagement can be fostered at the individual level, clinic or organizational level, and the policy level [2]. While promoting patient engagement in individual care through self-management is better understood, less is known about clinic or organizational level patient engagement [3]. Examples of clinic or organizational-level patient engagement include patients serving on patient advisory councils, becoming members of quality improvement committees, or participating in training staff [4]. These activities allow healthcare leadership to incorporate the patient perspective and care experience when considering new clinic initiatives, quality improvement projects, or community needs.

Working with patient advisors is a promising method for achieving ongoing system-level patient engagement that can integrate with clinic quality improvement initiatives and address patient experience of care. Patient advisors are patients who meet on a regular basis with clinic staff to improve care delivery at the clinic, hospital, or organizational level. In the United States, patient advisory councils are increasingly mandated within organizations to demonstrate a commitment to patient-centered care, such as the Patient-Centered Medical Home (PCMH) delivery model, which delivers team-based, data-driven and coordinated care [5]. Patient advisory councils are an optional criteria of the 2014 US National Committee for Quality Assurance (NCQA) Patient-Centered Medical Home recognition standards, which is a national accreditation program that awards PCMH certification [6]. Patient and family advisors are also a requirement within US Accountable Care Organizations, which are groups of doctors, hospitals, and other providers who coordinate spending in order to provide quality-based care for a designated group of patients rather than service-based care, in order to share in cost savings for reduced unnecessary care [7]. In the United Kingdom, patient involvement has a historical precedent of patient participation groups providing input for NHS primary care services since the 1970’s [8]. In Australia, trained consumer representatives sit on most medical committees [9] and consumer consultant positions have been developed in mental health services [10].

A strong ethical rationale, based on principles of equity and transparency, is often cited in efforts to improve patient engagement through programs such as patient advisory councils or input from patient representatives [11]. However, there has been a lag in the evidence base assessing for any objective benefits of patient engagement at the clinic or organizational level. A systematic review conducted in 2002 by Crawford et al. identified 42 papers published between 1966 and 2000, 31 of which were case studies [12]. Their analysis reported anecdotal findings that patient input at the clinic level seemed to improve readability of educational materials and improve staff attitudes towards patients; these studies lacked formal prospective or pre-post evaluation of patient advisory activities. Another systematic review examined the impact of public involvement in articles published from 1997 to 2009, but only focused on sites in the UK [13]. Our systematic review seeks to update Crawford et al.’s 2002 review to provide a current understanding of the impact of patient advisory councils on concrete healthcare outcomes with an international scope. Initiating and maintaining a patient advisory council or other forms of patient engagement requires a commitment of staffing and other resources, thus clinics and hospital leaders may be resistant to investing in patient engagement without clear evidence to support their benefit. Our primary aim is to investigate the impact of interventions involving patient advisory councils on clinical care outcomes, patient safety, and patient satisfaction, compared to care that doesn’t involve patient advisors, for participants at all healthcare settings. Our secondary aim is to survey the impact patient advisors have on healthcare changes such as priority-setting, patient materials, and impacts on patient advisors themselves.

## Methods

We conducted database search queries targeting articles involving patients serving in an advisory capacity in which authors mentioned clinical care, patient safety or patient satisfaction outcomes. This systematic review followed PRISMA guidelines (PRISMA checklist included in Additional file 1) and was registered in the PROSPERO database of the University of York Centre for Reviews and Dissemination (ID: 2015:CRD42015030020). The initial literature search was conducted from September–October 2015. Reference lists were further searched for relevant articles from October 2015 through May 2016.

### Identification of data sources

We searched PubMed, SCOPUS, CINAHL and Google Scholar databases. The search strategy included a combination of either patient engagement or patient advisory council terms as well as quality outcome terms [Table 1]; the initial PubMed search strategy was then adapted for SCOPUS, CINAHL and Google Scholar, with syntax and search specifications optimized for each search engine (see Additional file 2). Given that much of the work around patient engagement is shared in white papers or non-peer-reviewed publications, we also conducted grey literature searches in order to identify informally published or ongoing patient engagement research. Specific sources of grey literature included white papers available from the Institute for Patient and Family-Centered Care and the Patient-Centered Primary Care Collaborative. Reference lists of our final list of included studies were reviewed and abstracts of relevant articles were reviewed in a similar process. Identified studies were uploaded into DistillerSR, a web-based systematic review software [14].

**Table 1:** PubMed Search Strategy

(((Patient OR patients OR consumer OR consumers OR community OR communities) AND (“patient participation”[mh] OR “consumer participation”[mh] OR “patient engagement” OR “consumer engagement” OR “patient participation” OR “consumer participation” OR “consumer involvement” OR “patient involvement”))
OR
(“Advisory committees”[mh] OR “Governing board”[mh])) OR (“patient advisory council” OR “patient advisory committee” OR “patient and family advisory council” OR “consumer advisory council” OR “patient advisory committee” OR “consumer advisory boards” OR “Community advisory board” OR “Community advisory council” OR “Community advisory committee” OR “Community advisory boards” OR “Community advisory council”)
AND
(“Patient Harm”[Mesh] OR “Patient Safety”[Mesh] OR “Quality of Health Care”[Mesh] OR “Patient Satisfaction”[mh] OR “patient safety” OR “quality of care” OR “clinical outcomes” OR “patient experience” OR “patient satisfaction” OR “consumer satisfaction” OR “Community health planning” OR “population health” OR “Health Care Costs”[Mesh] OR “cost of care” OR “health care costs” OR “Health Priorities/organization and administration”[mh] OR “health care priority”[Tiab] OR “health care priorities”[Tiab] OR “healthcare priority”[Tiab] OR “healthcare priorities”[Tiab] OR “health priority”[Tiab] OR “health priorities”[Tiab] “Quality Improvement/organization and administration”[mh])

### Study selection

Two reviewers from the study team, one lead (AES) and one support researcher (VM or MK), independently reviewed titles and abstracts using DistillerSR. Included studies had to describe a patient advisory council activity or intervention, defined for the purpose of this study as a group of patients or consumers working with healthcare staff in order to provide input on healthcare services or delivery. Included studies also had to describe an impact on our primary or secondary outcomes of interest. Our primary outcomes of interest were clinical quality of care, patient safety, or patient experience of care. Our secondary outcomes of interest included other impacts of patient engagement activities to clinic services, policies, priorities, clinical physical space, or impacts on clinic staff or patient advisors themselves. We included randomized controlled trials, observational studies including cross-sectional surveys and qualitative assessments, and case studies. As the intention was to update the Crawford systematic review on patient engagement outcomes, the search query was limited to English language articles dated from November 2002 to August 2015. Inclusion criteria spanned all healthcare settings, including primary care, ambulatory specialty care, inpatient care, emergency department and long-term care. We excluded reports detailing patient engagement within their own individual care, patient engagement within research studies, as well as perspective, policy and protocol studies. A complete list of inclusion criteria is available in Table 2.

**Table 2:** Inclusion and Exclusion Criteria

Inclusion Criteria	Exclusion Criteria
Published between November 2002–August 2015	Published before November 2002 or after August 2015
English language	Non-English publication
Healthcare-focused; including but not limited to inpatient and outpatient, primary care, ambulatory sub-specialty, and emergency department	Study not located in a healthcare setting
Involves patient input on an advisory council, board, or committee	Did not involve patient input via a council, board or committee
Reports measure of patient engagement impact either for primary outcomes: clinical outcome measure or an NQMC safety or patient satisfaction measure for staff or patients; or secondary outcomes: other impacts on clinic processes, priorities, physical space or impacts on clinic staff or patient advisors themselves	Description of a patient engagement intervention that does not address primary or secondary outcomes.
Patient engagement activities described address organization/system-level changes	Exclusively addresses engagement in individual care such as shared decision making or patient activation processes; or involved patient engagement within a research protocol without patient engagement pertaining to the intervention itself
Is a research study or case report that includes assessment of patient engagement impact	Is a perspective, policy piece, or protocol.

Inclusion-exclusion conflicts were managed by direct discussion and review with an independent fourth senior researcher (NO). If there was a discrepancy, the full text was reviewed and discussed as a group until consensus was reached. Included abstracts were then reviewed in full-text data abstraction. Weighted overall kappa for abstract screen was 0.63 and for full text abstraction was 0.68, levels which indicates good agreement.

### Data extraction

Primary outcomes included clinical care quality measures as defined by the National Quality Measures Clearinghouse [15], patient safety measures and patient satisfaction measures reported by either clinical staff or patients and families. Secondary outcomes were other descriptions of patient advisory council impact on the healthcare setting. Both quantitative and qualitative markers of patient engagement effects were extracted using dual-review and an extraction form built in DistillerSR. We developed a data extraction form that collated study type, demographics of each study site (e.g., location, patient population, type of clinical practice), type of patient engagement intervention, outcome metric(s), a summary of the impact patient engagement had on that measure and possible study confounders, and limitations or conflicts of interest.

### Analysis

We applied a thematic analysis approach to synthesize review findings by type of patient advisory council and impacts based on prior systematic reviews that applied a similar approach to heterogeneous study results, rather than attempt a meta-analysis of aggregate data. Study quality was initially determined using the Cochrane Collaborative Bias Assessment Tool [16]. In order to assess quality for both qualitative and quantitative studies, we subsequently used the Mixed Methods Appraisal Tool, which rates studies on a scale from 0 to 4 (Additional file 3) [17].

## Results

**Fig. 1:**
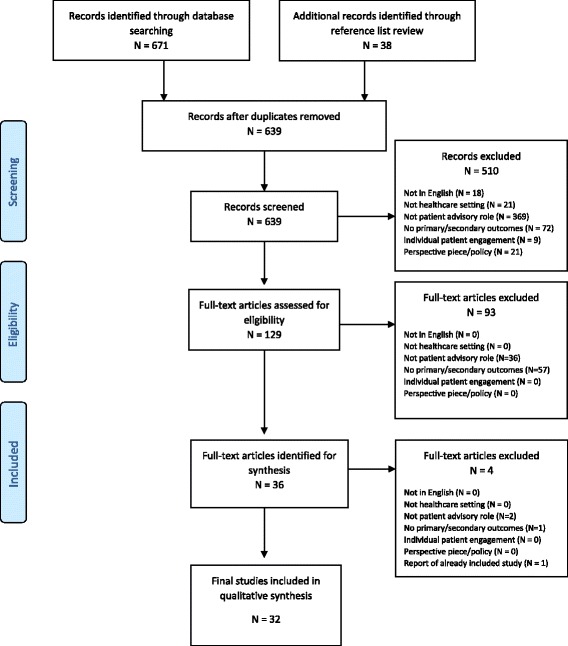
Prisma 2009 Flow Diagram

Database searching yielded 639 articles after duplicate removal. After abstract review, 129 articles were assessed for full-text data screening and abstraction. After full-text review, 93 articles were excluded yielding 36 articles. Of the 36 articles, one study was eliminated because it did not report our primary or secondary outcomes [18]. One study was eliminated because it was a brief report of a patient advisory council already described in detail in another included study [19]. Two studies were eliminated as they described community participation in regional public health governance in low-resource settings, without clearly referencing if patients were serving as advisors within a healthcare facility setting [20, 21]. We ultimately included 32 papers for final qualitative synthesis. A full PRISMA diagram of included studies is provided in Fig. 1.

Of included studies, 15 were based in the United Kingdom, 8 in the United States, 4 in Canada, 4 in Australia or New Zealand, and 1 in Sweden (see Table 3). One study was a cluster-randomized controlled trial, 4 described a set of quasi-experimental quality improvement initiatives from one practice group, 2 were systematic reviews, 1 was a cross-sectional survey, and 9 were qualitative or ethnographic studies. The majority (*N* = 16) were case studies comprising a description of a patient advisory project without formal quantitative, qualitative or ethnographic data included. The primary mode of patient advisor intervention was a patient advisory council, referring to a group of patients who met with staff on a regular basis to discuss healthcare improvement activities. Patient engagement was also described in studies as community advisory councils (4 from one institution), experience-based co-design projects, ad-hoc patient committees who met for a single project, and “other” activities such as “mental health user groups” composed of patients who advise healthcare trusts in the UK. Only one study qualified as having high quality and low risk of bias per the Cochrane Assessment tool [22]; the majority of studies were classified as “not applicable” for quality assessment due to their case-based nature or lack of prospective study design; eight of the studies scored the maximum 4/4 using the Mixed Methods Appraisal Tool. Full summaries of studies including design, intervention, findings and quality rating are included in Table 4.

**Table 3:** Categories of Included Articles

Type of Patient Involvement	N
Patient Advisory Council	11
Community Advisory Council	4
Ad-hoc Patient Committee	8
Experience-based co-design	4
Other	5
Article Type	
Randomized Controlled Trial	1
Quasi-Experimental Study	4
Systematic Review	1
Cross-Sectional Survey	1
Qualitative Study	9
Case Study	16
Location	
UK	15
US	8
Australia/NZ	4
Canada	4
Other (Sweden)	1
All Articles	32

**Table 4:** Summary of Studies Included in Systematic Review

Type of patient involvement	Author	Article type	Location	Description of Patient Engagement	Reported Effectiveness of Patient Engagement Intervention	Basis of Evidence	Quality Score (MMAT maximum score 4/4)
Community Advisory Council	Zittleman 2009; Bender 2011; Norman, 2013; Deaullme 2015	Quasi-experimental community awareness campaign	US	Community Advisory Council (17 members) of local farmers, ranchers, schoolteachers, students. Combination of in-person meetings, teleconferences, emails to review clinical guideline and plan "translation" to community. Also community focus groups and town halls.	Increased exposure to community message associated with increased intention to receive CRC screening. Increased use of controller inhalers, asthma action plans and spirometry in pre-post analysis. Improved blood pressure control.	Varied by study; pre- post- analysis comparing those exposed vs. non-exposed to the community intervention	Zittleman: Quantitative RCT 3/4; Bender: Quantitative non-randomized 4/4; Norman (N/A review paper); Deaullme: Quantitative nonrandomized 2/4
Patient Advisory Council (called Cancer Partnership Groups)	Richardson, 2005	Qualitative Study	UK	Average 75% cancer patients and 25% caregivers per group, usually meeting every 2 months	Focus group for new cancer center; networking with community groups; developing leaflets and "breaking bad news" training for providers; advocacy to improve support and access for cancer services	Telephone interviews with 27 patients from 34 cancer networks; site-based interviews with patients and staff at 6 sites	Qualitative 4/4
Patient Advisory Council	Bowen, 2004	Qualitative Study	Australia/NZ	"Consumer reference group" of 8-10 breast cancer patients meeting 4 times per year	Social connections and communication skills for patient committee members	Interviews with 9 members and staff	Qualitative 3/4
Patient Advisory Council	Kendell, 2014	Qualitative Study	Canada	15 members	Input on decisions but members unable to provide examples; Social connections for patient committee members, personal benefit of "feeling heard"	Semi-structured key informant interviews with patients, staff and community members (n=5)	N/A; did not pass screening criteria due to limited sample size
Patient Advisory Council	Perreault, 2010	Case Study	Canada	8-12 outpatient psychiatric patients and 4 staff members meeting 3-4 times per year	Mental health benefit for committee members, improved provider/staff awareness of patient experience, reduction of mental health stigma	Review of meeting agendas and projects, Group evaluation from panel members	Qualitative 4/4
Patient Advisory Council	McTavish, 2014	Case Study	Canada	Patient and Family Advisory Council (makeup not described) and 55 Patient experience advisors throughout the organization	Altered visiting hours, inclusion of patients on hospital committees, Discharge information, improved staff satisfaction, stable patient satisfaction, and tailoring services to patient needs. Trending although non-statistical increase in patient report "I have been listened to by healthcare team" and staff agreeing with having a collaborative practice	Case-based description, Inpatient pre- and post- evaluation survey for patients (N=624) and staff (398)	Quantitative descriptive 1/4
Patient Advisory Council	Rich, 2014	Case Study	US	18 members aged 12-19 at an academic children's hospital, meeting once per month, 11 months per year	Individual empowerment and advocacy skills, clinic culture, physical space, patient education tools	Case-based examples of projects	N/A; case study without formal evaluation
Patient Advisory Council	Loud, 2013	Case Study	UK	6 members with experience of long-term conditions, including CKD, diabetes, heart disease and kidney cancer; meeting 2-3 times per year as well as email and calls	Patient and staff educational materials to support chronic kidney disease self-management	Informal evaluation	N/A; case study without formal evaluation
Patient Advisory Council	White, 2012	Case Study	US	11 patient and family councils across different specialties/services; Executive Council of 8 patient advisors who sit on system-wide committees; serve 1-2 year terms	Change to clinic physical space, improved discharge process, improved scheduling, patient education materials, customer service training for staff, patient welcome video, improved billing statements	Case-based examples of projects	N/A; case study without formal evaluation
Patient Advisory Council	Ponte, 2003	Case Study	US	1 adult and 1 pediatric oncology patient advisory council	Access (e.g., an emergency department "fast track"), design of new physical space and plans for transferring patients to new space, new education program for first year oncology fellows	Case-based examples of projects	N/A; case study without formal evaluation
Patient Advisory Council	Meyers, 2008	Case Study (Grey literature)	US	Multiple sites described, one example: 60 to 70 advisors serving on more than 25 operational committees, including patient safety, education, ethics, grievance and hospital aesthetics	Potential reduction in falls and reduced error. Another site reports increased patient satisfaction (10 to 99th percentile), decreased length of stay (by 50%), increased discharge volume (by 15.5%), decreased medical errors (by 62%), and decreased staff vacancy (from 7 to 0%). Third site reported web portal development, physical improvements, patient-centered rounds, training medical students, input on research	Case study/Press release	N/A; case study without formal evaluation
Patient Advisory Council	Greenwood, 2003	Case Study (Grey literature)	UK	70 patients invited to provide feedback who had previously submitted complaints	Reduced patient complaints: informal complaints fell from 117 in 2 month period to 48 one year later; physical improvements: large-size x-ray gowns, higher chairs, less obtrusive bags for collecting belongings of deceased patients	Case study/Press release	N/A; case study without formal evaluation
Ad-hoc Patient Committee	Boivin, 2014	Cluster Randomized Controlled Trial	Canada	83 patients surveyed for input on primary care priorities; 17 patients worked with staff in 2 day deliberation session; patients sampled for age, gender, health status and SES	Healthcare services priority setting for improving chronic disease management in primary care	Priorities set with patient involvement in intervention arm were more aligned with PCMH and chronic care model (p<0.01)	Quantitative randomized 4/4
Ad-hoc Patient Committee	Forbat, 2009	Qualitative quasi-experimental study of QI intervention with control group	UK	3 lung cancer services worked with 10 patients and 3 family members on QI projects; 2 sites did not work w patient and were controls	Expansion of understanding of system-level patient involvement in intervention group compared to control; Improved relationship between patients and staff in intervention group	Pre- and Post-intervention focus groups with thematic analysis	Qualitative 2/4
Ad-hoc Patient Committee	Fudge, 2008	Qualitative Study/ Program Evaluation	UK	User involvement within a stroke care initiative in 2 boroughs over 2 years; included town-hall style forum to gather user input as well as ongoing working groups	Users provided input on questionnaire design, training materials for staff, educational materials including DVD for patients; trained to give peer support and raise community awareness. Users reported feeling listened to by staff and improved social relationships with other stroke survivors	Direct observation, semi-structured interviews and documentary sources	Qualitative 4/4
Ad-hoc Patient Committee	Anderson, 2006	Qualitative Study	UK	23 local residents interviewed; unclear total number involved in planning/design of Health Park and Health Center	Committee members contributed to planning new physical space, event publicity, individual empowerment, engagement of community members with governmental leadership	Individual interviews and focus groups	Qualitative 4/4
Ad-hoc Patient Committee	Robert, 2003	Qualitative Study	UK	Mental Health quality improvement collaborative across 37 NHS sites; involved at least one service user per site	Educational materials, patient record keeping, physical space (ward maps, photo boards), identified projects for PDSA cycles	Semi-structured interviews at 6 randomly selected case sites	Qualitative 4/4
Ad-hoc Patient Committee	Innes, 2003	Case Study	Australia/NZ	10 consumers reflecting diversity of residential area, ethnicity, age and breast disease status; meeting quarterly	Patient held record, newsletter, service reviews, participation in other breast cancer care committees, stronger relationships between committee members and staff	Focus group with consumer reference group and semi-structured interviews with senior executives	Qualitative 3/4
Ad-hoc Patient Committee	Carney, 2006	Case Study	UK	22 colorectal cancer patients (12 male; median age, 72 years, range, 40–86 years) who met three times	Created educational booklet	Case-based description of project	N/A; case study without formal evaluation
Ad-hoc Patient Committee	Ripley, 2007	Case Study	UK	Seven patient "users" with personal cancer history	Led familial cancer awareness presentations, contributed to educational leaflet, individual patient empowerment/social networking	Tally of monthly referrals to cancer screening service with qualitative increases after promotion months	N/A; case study without formal evaluation
Experience-based co-design	Piper, 2012	Qualitative Study/ Program Evaluation	Australia/NZ	169 patients/carers interviewed; 126 patients surveyed; in multiphase program involving staff across seven emergency departments total	Physical space, patient education materials, work flow changes to improve patient transfers/care coordination	Case-based description of projects and thematic analysis of interviews	Qualitative 3/4
Experience-based co-design	Tsianakas, 2011	Case Study	UK	23 breast and 13 lung cancer patients provided unstructured interviews that were filmed and edited to highlight areas for improvement. 37 breast and 26 lung cancer staff also interviewed. Staff/patient working groups then implemented changes based on data; unclear # of patients	Altered workflows to be more patient-centered; improved privacy in clinic spaces, improved appointment and scheduling access; improved patient education and group support; training for staff/trainees; decreased wait time for lab tests and appointments	Interviews, ethnographic fieldwork, interviews with participants after the project	Qualitative 3/4
Experience-based co-design	Boyd, 2012	Case Study	Australia/NZ	"Journey mapping" workshop of patients and their supporters (14), staff (5) and workshop organizers (2). 182 Experience-based survey completed (97 from breast clinic, 85 from mammography/ ultrasound)	Educational materials, patient record keeping systems, mammography gown design, patient-provider communication	Case-based description of project	Qualitative 3/4
Experience-based co-design	Gustavsson, 2014	Case Study	Sweden	New mothers and their partners (3 mothers, 2 fathers) collaborated with neonatal healthcare staff; patient and staff had focus groups	Recommendations for improving physical space and amenities (improved beds, meal service, alarm system), staff training, and communication	Case-based description of project	N/A; case description without formal evaluation
Other	Mockford, 2012	Systematic review	UK	Results included 28 studies describing patient involvement via NHS board membership, primary care boards and trusts.	Improved relationships between patients and health professionals, changes to physical space, educational materials, better awareness of healthcare services among some people	20 case studies, 5 evaluations, 1 survey, 2 secondary data analysis; none with measurement of impact of activities	Quantitative descriptive: 4/4
Other	Crawford, 2003	Cross-sectional Survey	UK	75 Mental health user groups from 17 trusts. User groups ranged from five to over 200 members (median 35); median levels of meeting attendance at meetings between 10-15 members	65% of trusts listed impacts including improvements to ward environments, organization of outpatient services and systems for supporting patients in crisis. Eight (47%) trusts reported user participation in planning meetings influenced service development and policies. Only 6/25 user groups reported being satisfied with user involvement.	User self-report in survey	Quantitative descriptive 4/4
Other	Sweeney, 2005	Qualitative Study/ Program Evaluation	UK	24 staff and 4 patients interviewed about project involving 4 hospital trusts	Improved communication with patients, enhanced staff attitude toward patient perspective, staff training, changes to clinical processes (such as discharge), involved patients felt "heard"	Individual interviews	Qualitative 4/4
Other	Challan, 2006	Case Study	UK	Clinical Audit (similar to QI) Patient Panel for a Primary Care Trust; 11 members	Panel conducted audit of pulmonary services and made recommendations; repeat audit 1 year later found improvements in: Access (Drop-in clinics offered and improved specialty referrals), patient self management information and support, education for staff	Case-based examples of projects	N/A; case study without formal evaluation
Other	Murie, 2004	Case Study	UK	Public health walk (670 people); 60 of which formed a community forum. Separate Patient Participation Group (started with 36 patients, decreased to 7 in 2 years) meeting monthly; mostly older retired professionals	Access (e.g., evening care, mental health teams), patient-held record card, new services (e.g., smoking cessation clinics, cardiac rehabilitation), co-located pharmacy, links to community transportation service	Case-based examples of projects	N/A; case study without formal evaluation

### Primary outcomes: Clinical care, patient safety, and patient satisfaction

#### Clinical care

No studies reported results from a prospective, randomized controlled trial with respect to our primary outcome of patient advisors impacting clinical care, patient safety, or patient satisfaction. We found four papers describing quasi-experimental public health interventions from one group in Colorado (United States) in which a community advisory council participated in a regional quality improvement campaign to publicize colon cancer screening, asthma, and blood pressure control within a practice-based research network. The community advisory council worked in an iterative process to “translate” health care promotions into public service messages that would be understandable to the lay community [23]. In accompanying articles, this approach was found to have positive results in several distinct interventions and was associated with statistically significant increased self-reported intention to engage in colorectal cancer screening [24], increased use of asthma inhalers as well as asthma action plans [25], and improved blood pressure control rates [26].

Six papers reported case-based or anecdotal findings that patient advisory councils had a role in improving appointment access for patients, which is a domain of care quality. In one case study, breast cancer patients in an experience-based co-design program recommended a change in the scheduling process for newly diagnosed patients [27]. Another case study described how patient and family advisory councils improved their hospital’s appointment scheduling so that the scheduling center contacted patients directly [28]. This change reportedly reduced the number of rescheduled appointments without a summary estimate provided. In the four remaining articles, patient advisory councils were involved in developing a "fast-track" system for an emergency department, improving public transportation to a clinic, and creating drop-in and evening clinic hours. [29–32].

#### Patient safety

One report included several case studies describing how patient advisory councils have had a role in patient safety. Examples included a United States hospital in Seattle, Washington that embedded patient advisory committees throughout the institution and attributed this participation to a reduction in falls and medical errors, however specific data on falls and medical errors were not reported. The report described another hospital initiative involving patient advisors in the redesign of their hospital, and attributed this redesign to a reduction in medical errors by 62% [33].

#### Patient satisfaction

Four papers described case-based results that patient advisory councils had a role in affecting patient satisfaction. One hospital’s mean patient satisfaction increased from the 10th to the 99th percentile; another hospital’s patient satisfaction scores climbed from 95% to 98%; these were both attributed to an investment in “patient-centered care” which included patient advisory councils as well as other programs [33]. In another article, a hospital system implemented patient advisory councils as part of a multi-pronged “Interprofessional Collaborative Practice Model” and performed pre-post analysis of patient satisfaction data in four units. The case study reported a positive trend in patient agreement that “I feel I have been listened to by the healthcare team,” although statistical analysis was not provided [34]. A patient and family advisory council in a pediatric oncology hospital in Boston MA implemented an ED “fast track” to expedite hematology-oncology admissions, which they attributed to an improved but unreported rate of patient satisfaction for oncology patients and their families [29]. A news article from a hospital in the UK shared how a hospital system trust invited patients who had submitted complaints to participate in regular meetings over two years to brainstorm improvements. Since implementing this program, complaints decreased from 117 to 48 in one year [35].

### Secondary outcomes: Clinic priorities, educational materials, physical improvements

#### Patients & health care priority setting

The most rigorous study found in this review was a cluster-randomized RCT based in Canada that compared an intervention arm, in which a random selection of regional primary care staff leaders worked with patients to identify priorities, against a control arm in which staff selected primary care priorities without patients. Priorities chosen by staff and patients working together were more aligned with the components of the Patient Centered Medical Home and the Chronic Care Model (*p* < 0.01), although the study could not assess whether these recommendations would be implemented by local primary care officials [22]. Six studies had case-based descriptions of how patient advisors helped to identify hospital or clinic priorities [12, 30, 31, 36, 37].

#### Benefits for patients and staff

Ten studies described case-based evidence that participation in an advisory council had a therapeutic or positive benefit for the patients themselves [8, 9, 36, 38–44]. A report of interviews with members of a children’s hospital teen advisory council found that participation fostered organizational skills and professional development [40]. A qualitative impact assessment of a panel of service users for a Montreal, Canada psychiatric institute interviewed the users serving in an advisory capacity. Users described improved mental health, enhanced education about services, and reduction of perceived stigma due to their involvement [36]. Five studies described how patient advisory councils increased hospital or clinic staff awareness of the patient perspective and patient-centered care [13, 36, 40, 43, 44].

#### Other impacts

Seventeen studies described how patient advisors helped develop materials for patient education or self-management [8, 9, 24–28, 30, 31, 40, 42, 45–50]. Fifteen studies described how patient advisors recommended changes to a healthcare setting’s physical space, such as an improved waiting room, improved accommodations for physical disabilities, or improved layout [13, 27–30, 32, 33, 35, 40, 41, 48–52]. Other studies described patient advisor involvement in workflow or service changes [27, 29, 30, 32, 34, 44, 52], patient-developed trainings for staff or trainees [27–31, 33, 42, 44, 49, 51], conference attendance, and web portal improvement [33].

One included study was another systematic review on the impact on patient involvement, but focused on care in the United Kingdom; the studies included in this review comprised narrative, case based evidence describing patient advisors contributing to our secondary outcomes such as improving physical space, expanding clinical services, devising educational materials, and changing healthcare staff attitudes or culture towards the patient perspective [13].

## Discussion

To our knowledge, this is the first systematic review with a focus on patient advisors, with an emphasis on quantifiable care quality outcomes. We did not find any rigorous, prospective RCTs that assessed our primary outcomes of patient clinical care, patient safety, or patient satisfaction. We did identify a group of quasi-experimental studies from one large regional initiative in which community advisory councils aided in the development of patient-centered messaging about the importance of colorectal screening, asthma and hypertension control. While the studies showed statistically significant improvement in health behaviors, the control group included those who did not encounter the health promotion program at all rather than a control health promotion message without patient input. We cannot definitively attribute the improvements in health outcomes seen to the patient engagement component. We found only one cluster-randomized trial showing patient advisors helped clinics set priorities that were better aligned with the PCMH and chronic care model.

Despite a growing policy focus on patient-centered care, research on organizational-level patient engagement has made little progress over the 15 years that have elapsed since the publication of the 2002 Crawford systematic review. Similar to the Crawford article, we observed the most commonly found examples of advisors having an impact were on improvements to patient educational materials, clinical physical space, and changes to staff “culture” or awareness. Our review included one other systematic review of patient and public involvement in the UK, which found very similar case-based evidence within our secondary outcomes. Our review adds to these findings through our international scope and ability to capture a variety of patient advisory roles. The few experimental studies did show improvements in some clinical care metrics, clinic priorities, and staff awareness of patient engagement; however more concrete outcome measures were lacking.

Objective clinical outcomes, including quality, safety, and patient satisfaction, should be assessed in order to provide a stronger evidence base for system-level patient engagement. It is likely a challenging environment to assess these domains, given that patient advisory councils are an incredibly heterogeneous intervention. Patient advisory councils typically work on a number of projects at a given time and there are rarely joint efforts for a similar project across multiple patient advisory councils or multiple sites. Additionally, implementation of an advisory council takes significant time and resources to recruit, hold meetings, and provide follow up [53]. Implementers of advisory councils likely lack the bandwidth to incorporate formal evaluations into their work.

In order to know if patient advisory councils are actually impactful, a future research agenda for patient engagement must overcome the limitations inherent to this field. Patient advisory councils and similar patient engagement approaches are ripe for knowledge translation approach, i.e., a close, interactive relationship between researchers and health systems to accelerate evidence and improve health systems [54], Individual sites working with advisory councils should incorporate quality improvement-informed data gathering tools and consider projects as Plan-Do-Study-Act cycles with iterative analysis of improvement. For example, a hospital could assess consumer satisfaction with a waiting room space before and after an advisory council-led renovation, or assess individual patients’ self-efficacy in managing diabetes before and after using a patient advisory council-devised diabetes pamphlet. Higher quality research will require concerted efforts across healthcare sites. For example, if a healthcare network implements patient advisory councils across multiple primary care sites, central leadership could encourage a shared quality goal (improvement in standardized patient satisfaction surveys, network-wide mammogram rates, etc.) which would provide an aligned outcome that could be assessed using either a pre-post analysis, stepped-wedge, or cluster-randomized design to provide a comparison group.

The study designs utilized by the few studies that did formally evaluate patient advisory councils can serve as a guidepost for future investigators interested in assessing the impact of patient engagement. The PBRN-based public health intervention partnered with community advisory council members, who were best suited for adapting and translating evidence-based health promotion materials so as to be compelling to community members [45]. If this intervention had included a study arm providing health promotion *without* community advisors, it would have allowed for a more rigorous analysis of how the community advisory council input improved effectiveness. In contrast, a robust control group was included in the cluster-randomized trial of an intervention working with patient advisors to set primary care priorities; by utilizing randomization, the study addressed the many potential confounders involved in clinic-level interventions. This study established a clear comparison group of staff working without patients, allowing the investigators to identify the changes in primary care priorities that could be attributed to patient involvement [22]. A comparison group was also included in a study assessing staff awareness of patient engagement before and after working with patient partners; qualitative analysis showed an expanded and improved understanding and receptivity to patient engagement in the study arm that worked with patient and family advisors [43]. By including a control arm and/or quantitative outcome measures, these studies highlight how patient advisors may enable healthcare programs to become more patient-centered.

As healthcare becomes more consumer-focused, we have much to learn from the business and industrial design world. The studies we identified exploring experience-based co-design are a promising start to incorporating the patient perspective in healthcare improvement efforts, although they did not include reports of pre- and post-evaluation of how changes affected the care experience, which would be helpful to understand if the co-design process should be widely disseminated.

Our study limitations include regional variations in terminology. In the US, “patient engagement” is the preferred term, while in the UK “patient and public involvement” is more common. “Co-creation” and “co-production” are growing in popularity in Australia [55]. While we may not have captured all of these variations with our search strategy, we designed a search strategy aimed to capture studies that included patients as advisors regardless of the “engagement” term used. Another limitation is possible confounding of results; some studies mentioned improvements in quality or patient satisfaction measures after large-scale care redesigns across a hospital that included patient advisory councils, making it less plausible that the improvements seen could solely be attributed to the work of the patient advisors. It is also likely that positive examples of patient advisory council projects are more likely to be written up than unsuccessful ones, thus the included case studies are an example of reporting bias. The group of studies that addressed our primary outcomes of interest were from one practice-based research network in Colorado, US, thus their findings were geographically bounded in one region. Finally, we may not have captured the most recently published studies relating to patient advisory councils.

Despite these limitations, our review highlights both the strengths and challenges inherent in the field of patient engagement research. While the currently published evidence base for patient advisors is limited and primarily case-based, it does provide a description of the scope of patient advisors roles and their potential for collaboration with staff on healthcare improvement efforts, which can serve as examples for staff beginning to work with patient advisory councils. The few studies that were RCTs or quasi-experimental should serve as references for future prospective evaluations.

## Conclusions

The studies included in this systematic review show how patient advisors have been involved in a wide range of projects oriented towards practice improvement. Patient advisors seem to help healthcare organizations develop programs that are more accessible or understandable which may translate to more effective primary care outcomes and priority setting. Case-based reports describe patient advisory council involvement in projects to improve clinical care and safety. Future work must seek rigorous evaluation of patient advisory council programs, in order to demonstrate value in the investments needed to implement patient-centered care.

## Additional files

Additional file 1: PRISMA Checklist. PRISMA is an evidence-based set of guidelines for reporting systematic reviews. The checklist confirms that authors have followed PRISMA guidelines when completing this systematic review. (DOC 63 kb)

Additional file 2: Search Syntax for All Included Databases. The supplementary table identifies how the search strategy was adapted to return focused results from each database included in the systematic review—PubMed, Google Scholar, CINAHL, and SCOPUS (DOCX 14 kb)

Additional file 3: Mixed Methods Appraisal Tool Scores. This supplementary table includes details on the scoring rationale for our quality assessment of our final included articles using the Mixed Methods Appraisal Tool (DOCX 109 kb)
