# Correction to: The impact of patient advisors on healthcare outcomes: a systematic review

**DOI:** 10.1186/s12913-018-3267-7

**Published:** 2018-06-11

**Authors:** Anjana E. Sharma, Margae Knox, Victor L. Mleczko, J. Nwando Olayiwola

**Affiliations:** 1 Department of Family and Community Medicine, Center for Excellence in Primary Care, UCSF, 995 Potrero Ave, Ward 83, San Francisco, CA 94110 USA; 2 Contra Costa Regional Medical Center, Family Medicine Residency Program, 2500 Alhambra Avenue, Martinez, CA 94553 USA; 3 University of California, San Francisco, 2120 University Avenue, Berkeley, CA 94704 USA

## Correction

Following publication of the original article [1], the authors reported that reference no. 4 be changed. The details of the correction are as follows:

Originally, reference 4 was stated as:

Willard-Grace RSA, Parker C, Potter MB. Engaging Patients as Partners in Practice Improvement: A Survey of Community Health Centers. J Clin Outcomes Manag. 2016;23(7):311–9.

The correct reference 4 is:

Willard-Grace, R., Sharma, A.E., Parker C, Potter, M.B. Engaging patients as partners in practice improvement: A survey of community health centers. JCOM. 2016;23(7):311–9.
