# Supplementary material for: The impact of patient advisors on healthcare outcomes: a systematic review
Source: BMC Health Serv Res. 2017 Oct 23;17:693. doi: 10.1186/s12913-017-2630-4 (PMC5651621; doi:10.1186/s12913-017-2630-4)
Supplement: Supplementary file 2 — Search Syntax for All Included Databases. The supplementary table identifies how the search strategy was adapted to return focused results from each database included in the systematic review—PubMed, Google Scholar, CINAHL, and SCOPUS (DOCX 14 kb) [file 12913_2017_2630_MOESM2_ESM.docx]

**The impact of patient advisory councils on healthcare outcomes: a systematic review**

*Search terms by database*

| Search | Terms |
| --- | --- |
| PubMed | (((Patient OR patients OR consumer OR consumers OR community OR communities) AND (“patient participation”[mh] OR “consumer participation”[mh] OR “patient engagement” OR “consumer engagement” OR “patient participation” OR “consumer participation” OR “consumer involvement” OR “patient involvement”))  OR  (“Advisory committees”[mh] OR “Governing board”[mh])) OR (“patient advisory council” OR “patient advisory committee” OR “patient and family advisory council” OR “consumer advisory council” OR “patient advisory committee” OR “consumer advisory boards” OR “Community advisory board” OR “Community advisory council” OR “Community advisory committee” OR “Community advisory boards” OR “Community advisory council”)  AND  ("Patient Harm"[Mesh] OR "Patient Safety"[Mesh] OR "Quality of Health Care"[Mesh] OR “Patient Satisfaction”[mh] OR “patient safety” OR “quality of care” OR “clinical outcomes” OR “patient experience” OR “patient satisfaction” OR “consumer satisfaction” OR “Community health planning” OR “population health” OR "Health Care Costs"[Mesh] OR “cost of care” OR “health care costs” OR "Health Priorities/organization and administration"[mh] OR “health care priority”[Tiab] OR “health care priorities”[Tiab] OR “healthcare priority”[Tiab] OR “healthcare priorities”[Tiab] OR “health priority”[Tiab] OR “health priorities”[Tiab] "Quality Improvement/organization and administration"[mh]) |
| Google Scholar | ((“patient participation” OR “consumer participation” OR “patient engagement” OR “consumer engagement” OR “patient participation” OR “consumer participation” OR “consumer involvement” OR “patient involvement”) OR (“Advisory committees” OR “Governing board” OR “patient advisory council” OR “patient advisory committee” OR “patient and family advisory council” OR “consumer advisory council” OR “patient advisory committee” OR “consumer advisory boards” OR “Community advisory board” OR “Community advisory council” OR “Community advisory committee” OR “Community advisory boards” OR “Community advisory council”))  AND  ("Patient Harm" OR "Patient Safety" OR "Quality of Health Care" OR “Patient Satisfaction” OR “patient safety” OR “quality of care” OR “clinical outcomes” OR “patient experience” OR “patient satisfaction” OR “consumer satisfaction” OR “Community health planning” OR “population health” OR "Health Care Costs" OR “cost of care” OR “health care costs” OR "Health Priorities/organization and administration" OR “health care priority” OR “health care priorities” OR “healthcare priority” OR “healthcare priorities” OR “health priority” OR “health priorities” AND "Quality Improvement/organization and administration") |
| CINAHL | (MJ+(((Patient OR Consumer OR User) AND ((Engagement OR Participation OR Involvement) OR (Advisory Committee OR Governing Board OR Advisory Council OR Advisory Board))) AND((Patient AND (Harm OR Safety OR Satisfaction OR Experience)) OR (Healthcare AND (priorities OR quality OR cost OR improvement))))) |
| Scopus | ( INDEXTERMS ( "patient participation"  OR  "consumer participation"  OR  "patient engagement"  OR  "consumer engagement"  OR  "patient participation"  OR  "consumer participation"  OR  "consumer involvement"  OR  "patient involvement"  OR  "Advisory committees"  OR  "patient advisory council"  OR  "patient advisory committee"  OR  "consumer advisory council"  OR  "patient advisory committee"  OR  "consumer advisory boards"  OR  "Community advisory board"  OR  "Community advisory council"  OR  "Community advisory committee"  OR  "Community advisory boards"  OR  "Community advisory council" )  AND  INDEXTERMS ( "patient care"  OR  "Patient Safety"  OR  "Quality of Health Care"  OR  "Patient Satisfaction"  OR  "patient safety"  OR  "quality of care"  OR  "clinical outcomes"  OR  "patient experience"  OR  "patient satisfaction"  OR  "consumer satisfaction"  OR  "Community health planning"  OR  "population health"  OR  "Health Care Costs"  OR  "cost of care"  OR  "health care costs"  OR  "Health Priorities"  OR  "organization and administration"  OR  "health care priority"  OR  "health care priorities"  OR  "healthcare priority"  OR  "healthcare priorities"  OR  "health priority"  OR  "health priorities"  AND  "Quality Improvement " )  AND  PUBYEAR  >  2001 ) |
