# Supplementary material for: The impact of patient advisors on healthcare outcomes: a systematic review
Source: BMC Health Serv Res. 2017 Oct 23;17:693. doi: 10.1186/s12913-017-2630-4 (PMC5651621; doi:10.1186/s12913-017-2630-4)
Supplement: Supplementary file 3 — Mixed Methods Appraisal Tool Scores. This supplementary table includes details on the scoring rationale for our quality assessment of our final included articles using the Mixed Methods Appraisal Tool (DOCX 109 kb) [file 12913_2017_2630_MOESM3_ESM.docx]

MMAT Assessments

August 2017

MMAT Tool

1. Zittleman: 3/4

Quantitative randomized controlled(trials)

2.1. Is there a clear description of the randomization (or an appropriate sequence generation)? YES

2.2. Is there a clear description of the allocation concealment (or blinding when applicable)? NO

2.3. Are there complete outcome data (80% or above)? YES

2.4. Is there low withdrawal/drop-out (below 20%)? NO (> 20% did not complete survey)

2. Bender

Quantitative nonrandomized: 4/4

3.1. Are participants (organizations) recruited in a way that minimizes selection bias? YES (all members of a PBRN, no other way to avoid that although may have bias in joining a PBRN)

3.2. Are measurements appropriate (clear origin, or validity known, or standard instrument; and absence of contamination between groups when appropriate) regarding the exposure/intervention and outcomes? YES

3.3. In the groups being compared (exposed vs. non-exposed; with intervention vs. without; cases vs. controls), are the participants comparable, or do researchers take into account (control for) the difference between these groups? YES

3.4. Are there complete outcome data (80% or above), and, when applicable, an acceptable response rate (60% or above), or an acceptable follow-up rate for cohort studies (depending on the duration of follow-up)? YES

3. Norman: this is review paper, N/A for MMAT screen as it was not detailed summary of findings. References were used to abstract outcomes.

4. Dellaume – quantitative non-randomized = 2/4 score

3.1: NO

3.2 YES

3.3 YES

3.4 NO

5. Loud. Looked at Thomas N and Loud F 2012: managing chronic kidney disease in primary care: a quality improvement study. For more details. Score is either n/a or 0/4 quantitative non-randomized.

6. Bowen 2004: Qualitative score: 3/4

1.1. Are the sources of qualitative data (archives, documents, informants, observations) relevant to address the research question(objective)? YES

1.2. Is the process for analyzing qualitative data relevant to address the research question (objective)? YES

1.3. Is appropriate consideration given to how findings relate to the context, e.g., the setting, in which the data were collected? YES

1.4. Is appropriate consideration given to how findings relate to researchers’ influence, e.g., through their interactions with participants?NO

7.McTavish 2014 Quantitative descriptive : 1/4

4.1. Is the sampling strategy relevant to address the quantitative research question (quantitative aspect of the mixed methods question)? NO (not discussed)

4.2. Is the sample representative of the population understudy? NO (not clear but presumed patients/providers from intervention sites)

4.3. Are measurements appropriate (clear origin, or validity known, or standard instrument)? YES

4.4. Is there an acceptable response rate (60% or above)? NO/DON’T KNOW

8. Perreault – qualitative: 4/4

1.1. Are the sources of qualitative data (archives, documents, informants, observations) relevant to address the research question(objective)? YES

1.2. Is the process for analyzing qualitative data relevant to address the research question (objective)? YES

1.3. Is appropriate consideration given to how findings relate to the context, e.g., the setting, in which the data were collected? YES

1.4. Is appropriate consideration given to how findings relate to researchers’ influence, e.g., through their interactions with participants? YES

9. Ponte: screening questions 0 (descriptive case study)

10. Boivin: quantitative randomized: 4/4

2.1. Is there a clear description of the randomization (or an appropriate sequence generation)? YES

2.2. Is there a clear description of the allocation concealment (or blinding when applicable)? YES

2.3. Are there complete outcome data (80% or above)? YES

2.4. Is there low withdrawal/drop-out (below 20%)? YES ( think)

11. Carney: screening questions=0 or N/A (case study description)

12. Innes: qualitative 3/4

1.1. Are the sources of qualitative data (archives, documents, informants, observations) relevant to address the research question(objective)? YES

1.2. Is the process for analyzing qualitative data relevant to address the research question (objective)? YES

1.3. Is appropriate consideration given to how findings relate to the context, e.g., the setting, in which the data were collected? YES

1.4. Is appropriate consideration given to how findings relate to researchers’ influence, e.g., through their interactions with participants? NO

13. Robert 2003 – qualitative 4/4

1.1. Are the sources of qualitative data (archives, documents, informants, observations) relevant to address the research question (objective)? YES

1.2. Is the process for analyzing qualitative data relevant to address the research question (objective)? YES

1.3. Is appropriate consideration given to how findings relate to the context, e.g., the setting, in which the data were collected? YES

1.4. Is appropriate consideration given to how findings relate to researchers’ influence, e.g., through their interactions with participants? YES

14. Gustavsson – report of EBCD process and findings, but objectives for evaluation not clear. Scores 0

15. Tsainakas 2011: Qualitative score 3/4

1.1: YES

1.2: YES

1.3: YES

1.4: NO

16. Mockford: Quantitative descriptive: 4/4

17. Sweeney 2005: Qualitative 4/4

18. Murie 2004: cannot find
